# Serological investigation and isolation of *Salmonella abortus equi* in horses in Xinjiang

**DOI:** 10.1186/s12917-024-03955-7

**Published:** 2024-03-15

**Authors:** Zhanhai Mai, Han Fu, Ronghao Miao, Chong Lu, Xiaosong Zhang, Ziwen Yuan, Peng Ji, Yongli Hua, Chen Wang, Yuhui Ma, Haifeng Deng, Yanming Wei

**Affiliations:** 1 College of Veterinary Medicine, Gansu Agricultural University, Lanzhou, China; 2 College of Veterinary Medicine, Xinjiang Agricultural University, Urumqi, China; 3 Animal Husbandry and Veterinary Station of Bazhou Center for Disease Control and Prevention of Korla, Korla, China; 4 Animal Husbandry and Veterinary Medicine Development Center of Zhaosu County, Zhaosu, China; 5 Zhaosu Horse Farm, Ili Kazakh Autonomous Prefecture, Zhaosu, China

**Keywords:** *Salmonella abortus equi*, Serological investigation, *FliC*, *InvA*, Drug sensitivity, Equine

## Abstract

**Background:**

*Salmonella enterica subspecies enterica serovar abortus equi* (*S. abortus equi*) is one of the main pathogens that causes abortion in pregnant horses and donkeys, which was highly infectious and greatly restricts the healthy development of the horse industry.

**Objectives:**

In order to investigate the prevalence and biological characteristics of *S. abortus equi* in different regions and breeds of horses in Xinjiang.

**Methods:**

This study conducted ELISA detection of *S. abortus equi* antibodies on serum samples of 971 horses collected from three large-scale horse farms and five free-range horse farms in Yili Prefecture and Bayingol Mongolian Autonomous Prefecture of Xinjiang from 2020 to 2023. On this basis, bacterial isolation, culture, identification, and drug sensitivity tests were conducted on 42 samples of aborted foal tissues and 23 mare vaginal swabs.

**Results:**

The results showed that the positive rate of *S. abortus equi* antibody was as high as 20.91% in 971 horse serum samples. Among them, the positive rate in the Ili region (29.09%) was significantly higher than that in the Bayingole region (11.24%), and the positive rate in mares (22.45%) was higher than that in stallions (14.05%). In terms of horse breeds, the positive rates of self-propagating thoroughbred horses, half-bred horses, Ili horses and Yanqi horses were 43.22%, 28.81%, 14.72% and 11.24% respectively. In addition, *S. abortus equi* was more susceptible to juvenile and elderly horses, with positive rates of 70.00%and 41.86%, respectively, both of which were significantly higher than young (10.97%) and adult (19.79%) horses. Further, 9 strains of *S. abortus equi* were obtained through bacterial isolation, culture and identification, which were resistant to five antibiotics (Clarithromycin, Clindamycin, penicillin, Sulfamethoxazole and Rifampicin), and sensitive to 13 antimicrobial agents (Amoxicillin, Ciprofloxacin and Gentamicin, et al.).

**Conclusion:**

There was a high infection rate of *S. abortus equi* in Ili Prefecture and self-propagating thoroughbred horses, and juvenile or old mares were more susceptible, which will provide scientific basis for the prevention of *S. abortus equi* infection in different regions and breeds of horses in Xinjiang.

**Supplementary Information:**

The online version contains supplementary material available at 10.1186/s12917-024-03955-7.

## Introduction

In recent years, equine paratyphoid caused by *Salmonella enterica subspecies enterica serovar abortus equi* (*S. abortus equi*) has emerged as an important infectious disease that hinders the rapid growth of the horse industry as the horse breeding industry develops swiftly in Xinjiang, which has inflicted significant economic losses on the horse industry [1]. *S. abortus equi* only attacks equines, which can cause abortion and endometritis in pregnant mares, orchitis and swollen joints in stallions, and diarrhea, septicemia, and bronchopneumonia in young foals [2–4]. The Salmonella invasion gene A (*invA*) encodes proteins required for adsorption and invasion of epithelial cells [5, 6]. *invA* is frequently used to identify Salmonella since it is a virulence gene shared by bacteria of the genus Salmonella, with a highly conserved sequence [7–9]. This disease is distributed worldwide. As reported, large numbers of horses suffered from abortions in Europe and the United States as early as the late 18th and early 19th centuries, and equine abortus salmonellosis also successively broke out in northern, northwestern, and northeastern China in the late 1970s. Equine abortus salmonellosis can occur all year round, especially in spring and autumn, and majorly occurs late in pregnancy, which is mostly epidemic and sometimes endemic. Moreover, primiparous mares and foals are the most susceptible to this disease [10]. Most mares can recover spontaneously from abortion and secondarily develop diseases such as endometritis and vaginitis in a few cases. The pathogenic bacteria are excreted with the aborted fetuses, meconium, amniotic fluids, and vaginal secretions for pregnant mares following abortion and with semen for diseased stallions [1]. *S. abortus equi*-induced horses are usually carriers of the bacterium and are also often considered as the initial sources of infection in uninfected areas [11]. Although it was strictly controlled in European countries and the United States, the disease has not been fully controlled in African and Asian countries and has resulted in incalculable economic losses to equine breeders in endemic areas [12]. Previously, we did not understand the circulating characteristics of *S. abortus equi* in the 2 regions, as well as the physiological characteristics and drug resistance of the bacteria so far, therefore, in this study, by using 971 equine sera, we learned about the infection situation of *S. abortus equi* in Xinjiang and bacterial resistance, so as to better provide the basis for the comprehensive prevention and control of *S. abortus equi* in the future.

## Materials and methods

### Sample collection

A total of 971 blood samples were collected with the multi-stage random sampling method from horses (including Ili horses, self-propagating thoroughbred horses, half-bred horses, and Yanqi horses) of 8 large-scale horse farms in Ili Prefecture and Bayingol Mongolian Autonomous Prefecture, Xinjiang, between June 2021 and January 2023. None of the tested horses had a history of vaccination. Serum was prepared through centrifugation in batches and stored at -80℃ for centralized testing. Meanwhile, 23 samples of vaginal swabs and 42 aborted fetuses were obtained from two large-scale horse farms in Zhaosu County with a high rate of abortion.

### Serological detection for *S. abortus equi*

Serological detection was performed according to the instructions of the enzyme-linked immunosorbent assay (ELISA) kit (Harbin Veterinary Research Institute, Chinese Academy of Agricultural Sciences) for *S. abortus equi*. Specifically, the serum was diluted at 1:200 and maintained at 37℃ for 45 min. After the liquid in the wells was discarded, the samples were washed 4 times and cultured with ELISA antibody at 37℃ for 30 min. Subsequent to 4 washes, the samples were colored with the tetramethylbenzidine solution at 37℃ for 10 min, which was terminated with 2 mol/L H_2_SO_4_. Negative and positive controls were set up. The results were considered positive if the S/P value was ≥ 0.30 and negative if the S/P value was < 0.30.

### Histopathology

Tissue specimens of heart, liver, spleen, lung and kidney from the necropsied fetuses were collected and fixed in 10% neutral buffered formalin solution. From each placenta, multiple samples encompassing the areas showing gross changes were collected and fixed in the same fixative. After 2 weeks, when the fixation period was completed, the tissues were trimmed, dehydrated in ethanol and chloroform, and embedded in paraffin. Thin Sect. (4 μm) were prepared from paraffin blocks and consecutively stained with haematoxylin and eosin. All prepared sections were studied microscopically, and photographs taken of the lesions of interest.

### Isolation, purification, and morphological observation of bacteria

The aseptically collected vaginal swabs and aborted fetal tissue samples were clipped, inoculated in a liquid medium for pre-enrichment and incubated at 37℃ and 180 rpm for 12–16 h. Afterward, the inoculation loop was utilized to dip the bacterial solution on salmonella-shigella (SS) solid medium for plate streaking, followed by 16–20 h of inverted culture. The morphology of the colonies was observed, and single colonies were selected and cultured in a liquid medium at 37℃ and 180 rpm for 12–16 h for purification. Purification cultures of the same colonies of morphologically similar, this procedure was repeated three times, and bacterial Gram staining microscopy was performed to observe the morphology of the bacteria until all of the colonies on the medium were found.

### Biochemical identification of bacteria

Based on the Berger’s Manual of Systematic Bacteriology, the casual two strains of purified bacteria were inoculated in urease, sorbitol, mannitol, lysine decarboxylate broth, potassium cyanide, salicin, lysine decarboxylate control, malonate, indole (tryptophan broth), o-nitrophenol-β-D-galactoside (ONPG; β-galactosidase), potassium cyanide control, and dulcitol semi-solid microbiochemical identification tubes for 24–48 h of incubation at 35 °C.

### Amplification and sequence analysis of 16 S rRNA gene, *FliC*, *invA* and *FljB*

Based on the sequences of Salmonella *FliC* and *invA* genes in GenBank, two pairs of specific primers were designed respectively with the Primer Premier 5.0 software and synthesized by Sangon (Shanghai, China). The primer sequences are depicted in Table 1. The PCR reaction system was 12.5 µL, including 6.5 µL of Taq DNA polymerase, 1 µL of DNA template, 0.5 µL of forward and reverse primers each, and ddH_2_O that was added to 12.5 µL. The reaction conditions were as follows: pre-denaturation at 95℃ for 5 min, 36 cycles of denaturation at 95℃ for 30 s, annealing at 56℃ for 30 s, and extension at 72℃ for 90 s, and total extension at 72℃ for 10 min (PCR for 16 S rRNA gene); 95℃ pre-denaturation for 3 min, 34 cycles of denaturation at 95℃ for 30 s, annealing at 52℃ for 30 s, and extension at 72℃ for 30 s, and total extension at 72℃ for 10 min (double PCR for *FliC* and *invA* genes); 94℃ pre-denaturation for 5 min, 35 cycles of denaturation at 94 ℃ for 30 s, annealing at 62 ℃ for 30 s, and extension at 72 ℃ for 2 min, and extension a72°C for 10 min (PCR for *FljB* gene).

**Table 1:** Primers used in PCR to detect targeted genes

Gene	Primer oligonucleotide sequences (5′-3′) (forward/reverse)	Amplicon size (bp)	Annealing temperature (℃)
*16 S*	AGAGTTTGATCCTGGCTCA/ACCTGTCACCCGATGTACC	1460	56
*FliC*	CCAGACTCAGTTCAACGG/AAACCGCCATCAATAGTC	588	52
*invA*	GTCACCGTGGTCCAGTTT/CTCTTTCCAGTACGCTTCG	175	52
*fliB*	GTAGGATCCTCCATTFGCGCGCAGACCACTGAAGGCGC/ TAACTCGAGTTTCGGTCGCGTAGTCGGAATCTTCGA	1195	62

The PCR products were detected through gel electrophoresis, and the samples with positive results were selected for gel recovery. The gel-recovered products were sent to Sangon. After the sequencing results were analyzed with the Basic Local Alignment Search Tool (BLAST), the DNA Star software was used for the sequence and similarity analysis of the nucleotide sequences of the isolated strains with similar strains uploaded in GenBank, and phylogenetic trees were constructed with the Mega 7.0 software.

### Drug sensitivity test

Based on the constructed phylogenetic tree, the K-B disk diffusion method was utilized to select the two isolated strains of distant relationship, XZJ-L5 and XZJ-L10, followed by the analysis of their sensitivity to amoxicillin, cefuroxime, ciprofloxacin, clarithromycin, clindamycin, doxycycline, levofloxacin, gentamicin, cefoxitin sodium, penicillin, tetracycline, sulfamethoxazole, rifampicin, norfloxacin, methicillin, ampicillin, enrofloxacin, ceftriaxone sodium, and streptomycin. The culture solution (80 µL) of the isolated strains was spread evenly on the nutrient agar plate, and the drug sensitivity discs were placed in the corresponding positions of the plate in turn and pressed gently with forceps to stick to the solid medium evenly. Thereafter, the plate was placed in a 37℃ incubator overnight, and the diameter of the bacteriostatic circle. The sensitivity of the isolated strains to antibiotics was determined according to WS/T125-1999 standard, and the results were expressed as sensitive (S), intermediary (I), and resistant (R) [13].

### Growth curve

The positive isolated strains stored at -80℃ were thawed gradually, after which 100 µL of the frozen solution was inoculated into 10 mL of liquid medium, and the disposable culture tubes were placed in a constant temperature shaker at 37℃ and 180 rpm for culture, and then the samples were taken out every 2 h and added into the ELISA plate. The optical density (OD) values at 600 nm were measured in parallel three times and averaged. The growth curve was plotted with the OD value as the vertical coordinate and the culture time as the horizontal coordinate.

### Tertiary structure prediction of *FliC* protein in nine strains of *S. abortus equi*

The tertiary structure of *FliC* flagellin was predicted from nine amino acid sequences of isolates XJZ-L1-XJZ-L9 based on SWISS-MODEL (http://swissmodel.expasy.org) homology modelling online analysis, in which the similarity value was above 40%, indicating that the to-be-predicted protein and the template protein structure may belong to the same family, i.e., and considered as the homologous proteins. Then the results of homology modelling were evaluated according to the GMQE value and QMEAN value, and selected the prediction model whose GMQE value was between 0 and 1 (the closer to 1, the better the quality of modelling), and the QMEAN value interval was between − 4 − 0 (the closer to 0, the better the match).

### Data statistics and analysis

Statistical analysis was performed with SPSS 26.0, and the chi-square test was used for analyzing the positive rate of *S. abortus equi* infection in horses of different breeds, genders, and ages. *P* < 0.05 represented a significant difference. All figures and tables were generated with Adobe Photoshop Album 21.0.1 (Adobe Systems Software Ireland Ltd., Dublin, Ireland) and GraphPad Prism 9.0 (GraphPad Software, San Jose, CA, USA).

## Results and discussion

### Comparation of *S. abortus equi* antibodies in horse serum from different regions, breeds, ages, genders and feeding methods

#### Different regions

**Fig. 1:**
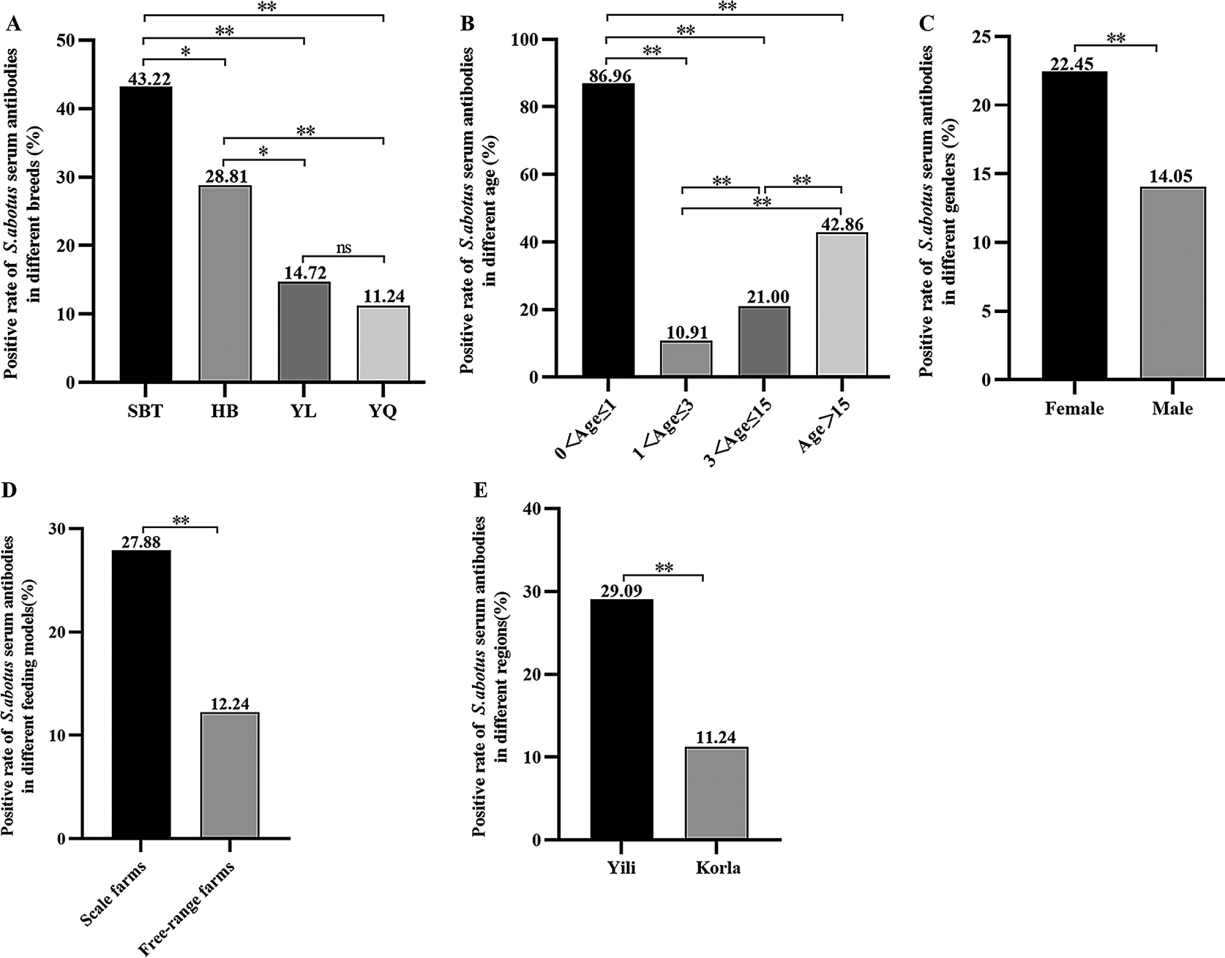
Positive rate of *S. abotus equi* serum antibodies in different breed, age, genders and feeding models. (**A**) The detection results of *S. abotus equi* serum antibodies in different breed; (**B**) The detection results of *S. abotus equi* serum antibodies in different age; (**C**) The detection results of *S. abotus equi* serum antibodies in different genders; (**D**) The detection results of *S. abotus equi* serum antibodies in different feeding models; (**E**) The detection results of *S. abotus equi* serum antibodies in different regions. Significant differences (*P* < 0.05) are indicated as “*” in the graph, and highly significant differences (*P* < 0.01) are shown as “**” in the graph, the same as follows

The 971 blood samples from eight large-scale horse farms contained 203 positive samples, with a mean positive rate of 20.91%. The mean positive rate was 29.09% (153/526) in horses from three large-scale horse farms in the Ili region and 11.24% (50/445) significantly higher than in horses from five large-scale horse farms in the Bayingol region (*P* < 0.01) (Fig. 1E and Supplementary Table 1). The highest positive rate was 41.79% (117/280) in horses from horse farm A of Zhaosu County in Ili, followed by 23.53% (20/85) in horses from horse farm C. A significant difference was found in the positive rate between Ili and Bayingol regions (*P* < 0.01) (Supplementary Table 2). Conducted an epidemiological survey of *S. abortus equi* infections in horses from Xinjiang in the period of 2013 to 2014 [14]. The results elucidated that the antibody positive rate of the bacterium in serum of horses was 28.28%. Yuan et al. [15] carried out a survey and study on the prevalence of *S. abortus equi* infections in horses from Ili, Xinjiang, and found an antibody positive rate of the bacterium of 25.31%. This result illustrated that the infection rate of *S. abortus equi* was continuously elevated in the Ili region and markedly lower in horses from the Bayingol region than in the Ili region.

#### Different breeds

The antibody positivity rate was the highest in self-propagating thoroughbred horses (43.22%), followed by half-bred horses (28.81%), Ili horses (14.72%), and Yanqi horses (11.24%) (Fig. 1A and Supplementary Table 3) (*P* < 0.01). This suggests that self-bred thoroughbred horse was more susceptible to *S. abortus equi* compared to Yil and Yanqi native horses, the lowest positive rate of *S. abortus equi* antibodies in serum from Yanqi horses was largely attributed to the fact that Yanqi horses were mainly herded because of their durable and hardy characteristics. Meanwhile, horses with purer bloodline are more susceptible to *S. abortus equi* infection.

#### Different ages and genders

The age of horses in the eight farms ranged from 0 to 22 years and could be categorized into four stages: juvenile (≤ 1 year), youth (1–3 years), adult (4–15 years), and old age (> 15 years), with the highest stock of adult horses. Horses at the juvenile stage exhibited the highest positive rate of *S. abortus equi* antibodies (70%, 21/30), of which mare foals had a positive rate of 40% (12/30). The lowest positive rate of *S. abortus equi* antibodies was found in horses at the young stage (10.97%, 17/155), with a markedly higher positive rate in young mares than in stallions (*P* < 0.01). (Fig. 1B, Supplementary Tables 4 and 6). The positive rate of *S. abortus equi* antibodies was substantially higher in mares (22.45%, 178/793) than in stallions (14.05%, 25/178) (*P* < 0.05) (Fig. 1C and Supplementary Table 6). The higher infection rate in mares than in stallions suggested that mares are more susceptible to *S. abortus equi* infections and that the stock of mares on farms is higher than that of stallions. Accordingly, mares should be immunized to prevent the disease. Reportedly, *S. abortus equi* infections widely occur in horses of all ages [15]. The older ages were associated with higher susceptibility to *S. abortus equi* infections, concordant with our results showing an age-dependent increase in infection rates. Our data displayed the highest positive rate of *S. abortus equi* antibodies in horses under 1 year of age, which can be explained by the fact that foals aged from 5 to 6 months acquired gradually decreased maternal antibodies and weaker autoimmunity, which results in a greater susceptibility to *S. abortus equi* infections of these horse than horses at other ages. Therefore, it is necessary to strengthen the feeding management and vaccination of foals at this stage.

#### Different feeding methods

Analysis of serum from large-scale and free-range horses demonstrated a positive rate of *S. abortus equi* antibodies of 27.88% (150/538) and 12.24% (53/433) for large-scale and free-range horses, respectively (*P* < 0.01), with significant differences between the two groups (Fig. 1D and Supplementary Table 7). The positive rate was extremely significantly higher in horses from centralized large-scale farms when compared with that in grazing free-range horses, indicating that excessive rearing density is not conducive to the prevention and management of *S. abortus equi* infections. Hence, rearing density should be emphasized for intensive large-scale feeding of horses. Meanwhile, diligent disinfection, epidemic prevention and quarantine should be highlighted.

In summary, *S. abortus equi* infection still exists in some areas of Xinjiang, and self-bred thoroughbred mares were more severely infected compared to Yanqi mares, with young foals and older horses being more susceptible to the disease.

### Autopsy examination of fetuses at different ages

**Fig. 2:**
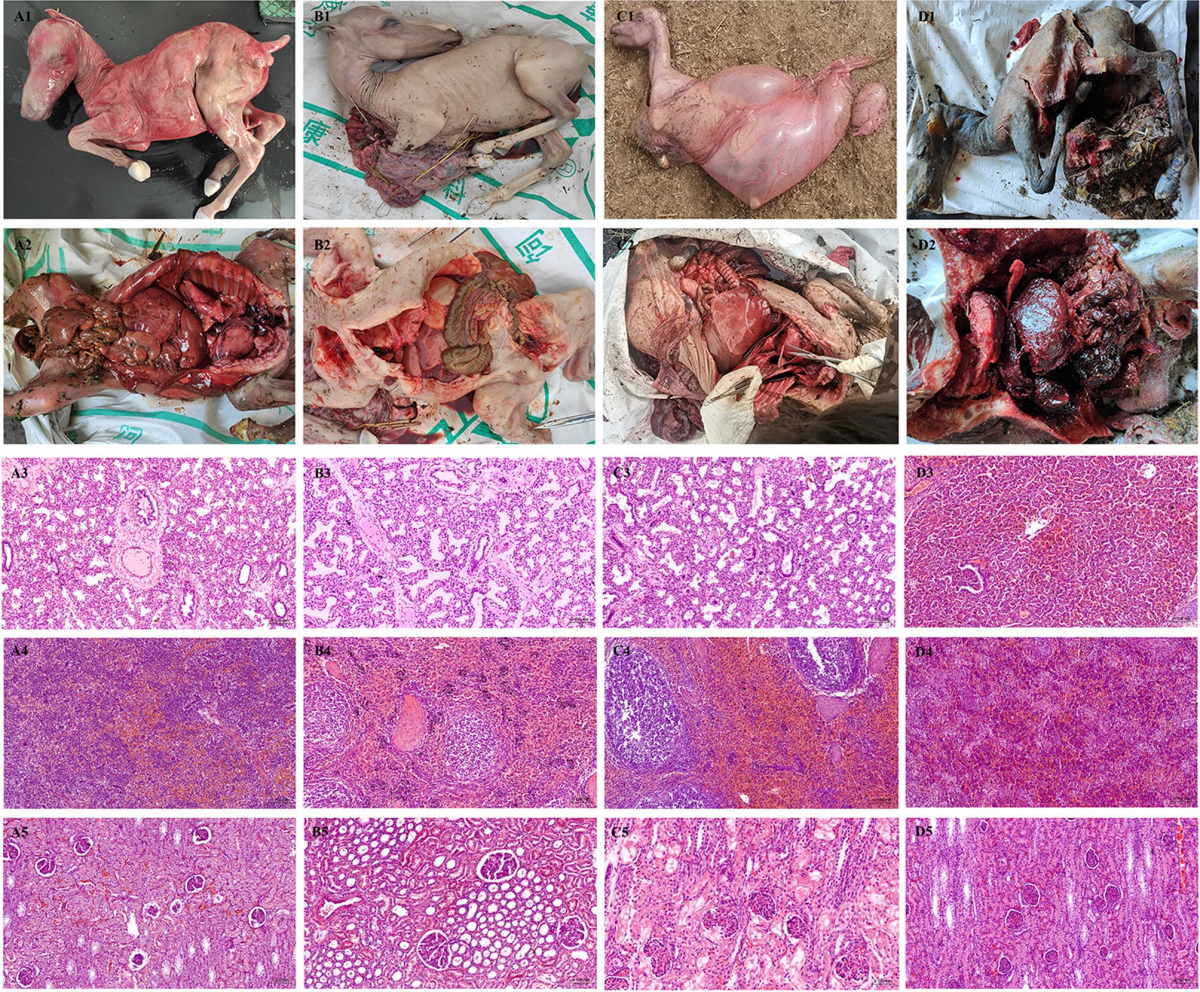
Different age Fetal autopsy examination. Fetuses with different ages (A1, 5 months; B1, 6 months; C1, 7 months and D1, 8 months) and signs of septicemia and friable organs of varying degrees at necropsy (A2, B2, C2 and D2); Interstitial inflammatory cell infiltration, interstitial widening, alveolar collapse, and atrophy in the lung (A3, B3, C3 and D3) (Hematoxylin and eosin, bar, 100 μm, 200×, the same below); Diffuse hemorrhage of splenic lymphocytes with localized foci of necrosis and increased ferrous hemosiderin (A4, B4, C4 and D4); Congestion of the kidneys (A5, B5, C5 and D5)

Abortion in the Zhaosu area of Ili, Xinjiang occurred from 5 months to 8 months of gestation, particularly in the second trimester of pregnancy (Fig. 2A1-D1). Signs of sepsis and organ fragility of varying degrees were observed at pathological anatomical (Fig. 2A2-D2). Interstitial inflammatory cell infiltration, interstitial widening, alveolar collapse, and atrophy in the lung (Fig. 2A3-D3). Diffuse hemorrhage of splenic lymphocytes with localized foci of necrosis and increased ferrous hemosiderin (Fig. 2A4-D4). Congestion of the kidneys (Fig. 2A5-D5). In general, most mares manifest no obvious clinical signs before the occurrence of induced abortion. For sudden abortion, most aborted fetuses are stillborn, and even a few surviving fetuses also die several days after birth [16]. Previous research unraveled that abortion in mares induced by this disease generally accounted for 30-60% of all abortions and once *S. abortus equi* was introduced in clean areas, the abortion rate was up to 90% or more. The gestation of mares lasts for about 11 months [17], and abortion in pregnant horses mainly occurs around the 5th to 9th month of gestation, suggesting that middle gestation is key for abortus equi infections and findings the sepsis and organ fragility in all aborted fetuses [3, 4].

### Isolation, purification, and identification of bacteria

**Fig. 3:**
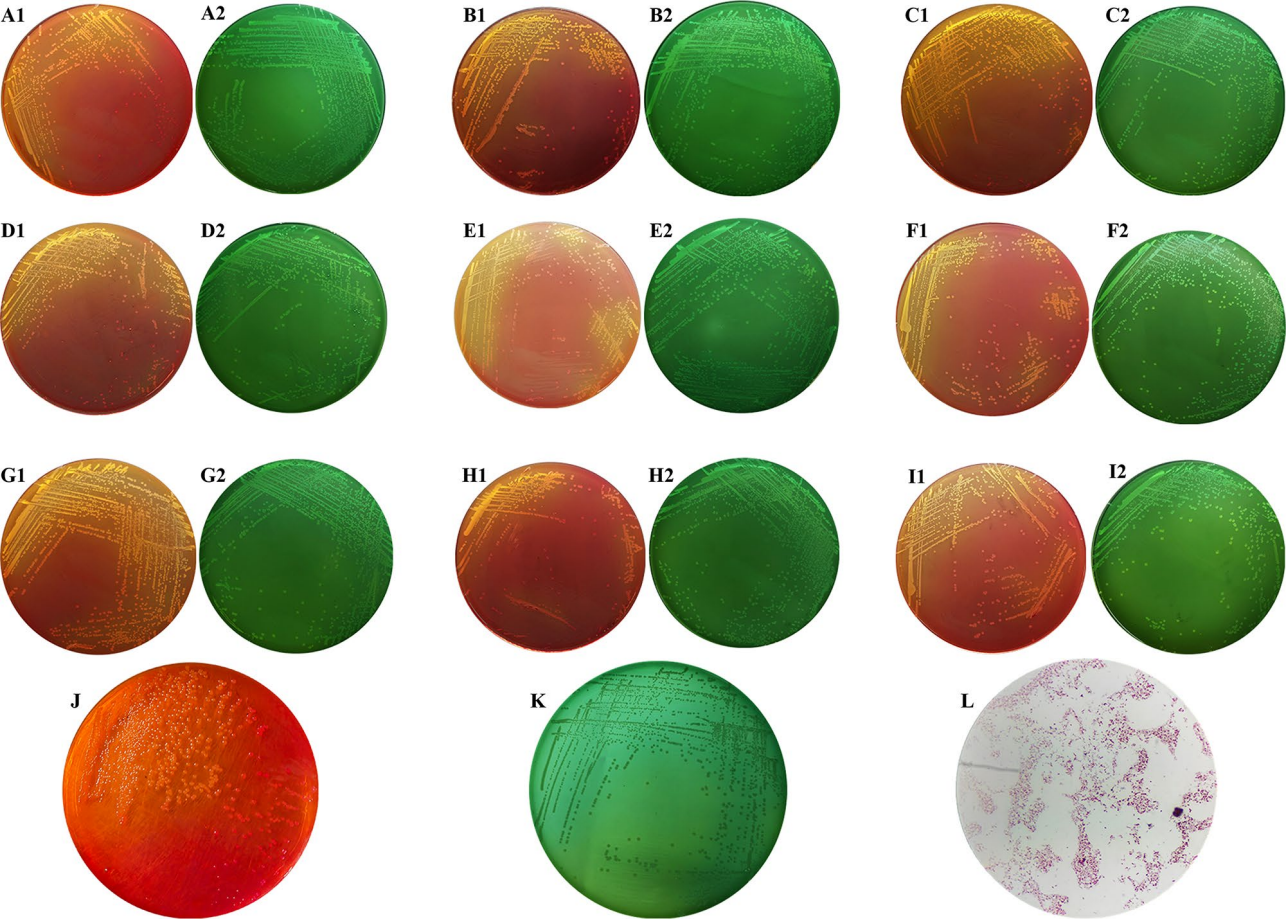
Culture and morphology characteristics of isolated strains. (A1-I1) are the morphology of XJZ-L1-9 9 strains on SS medium and HE medium (A2-I2), respectively; (J) Colony morphology on SS medium; (K) Colony morphology on HE medium; (L) Morphology of isolated strains after Gram staining (400×)

The 65 fetal tissue samples and vaginal swabs from aborted mares were subjected to culture and identification of pathogens. Of note, 9 strains of suspected were isolated, with a positive rate of 14% for the samples. The morphology of colonies formed by the nine isolates on the SS agar medium is detailed in Fig. 3A1-I1, and that on the HE agar medium is displayed in Fig. 3A2-I2. In summary, all the isolated bacteria were medium-sized and round, which appeared as protuberant, colorless, and round colonies on the SS agar medium (Fig. 3J) and as smooth and moist blue-green colonies with neat edges and no black centers on the HE agar medium (Fig. 3K). The purified bacteria were further identified with Gram staining, and the microscopic examination results unveiled that these bacteria had no capsule, no spore, bluntly rounded ends, and were uniformly dispersed Gram-negative brevibacterium (Fig. 3L). Subsequently, systematic pathogenic testing was conducted with visceral tissues of aborted foals and vaginal swabs of aborted mares in Ili, Xinjiang, which finally identified *S. abortus equi* infection in horses, suggesting that *S. abortus equi* infections have become endemic in Xinjiang, China, and timely preventive and therapeutic measures are required. NY-T570-2002 Diagnostic Techniques for *S. abortus equi* clearly states that the bacteriological test is the basis for the diagnosis of equine abortus salmonellosis [10].

### Biochemical identification results of bacteria

The biochemical test results showed that nine isolates reacted positively to urease, sorbitol, mannitol, lysine decarboxylate broth, potassium cyanide, salicin, lysine decarboxylate control, malonate, indole, and dulcitol semi-solids but responded negatively to ONPG and potassium cyanide control. The nine isolates were consistent with the biochemical characteristics of *S. abortus equi*. The biochemical reaction results of the isolated strains are shown in Table 2.

**Table 2:** Biochemical identification results of isolated strains

Item	Results	Item	Results
urease	+	malonate	-
sorbitol	+	salicin	+
mannitol	+	lysine decarboxylate control	+
ONPG	-	malonate	+
lysine decarboxylate broth	+	Indigo substrate	+
potassium cyanide	+	Dulcitol Semisolid	+

+, Positive; -, Negative

### 16 S rRNA, *FliC, invA*, and *FljB* amplification and phylogenetic analysis

**Fig. 4:**
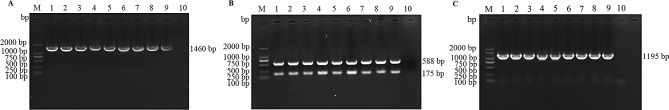
PCR amplification results of 16 S RNA, *FliC*, *invA and FljB* genes of isolated strains. (**A**) PCR amplification results of 16 S RNA genes of isolated strains; (**B**) PCR amplification results of *FliC*, *invA* genes of isolated strains (M, DL 2000 DNA Marker; 1–9, XJZ-L1-9; 10, Negative control); (**C**) PCR amplification results of *FljB* genes of isolated strains (M, DL 2000 DNA Marker; 1–9, XJZ-L1-9; 10, Negative control)

The 16 S rRNA gene was amplified through PCR with the genomic DNA of the nine isolated strains as the template, yielding a target band of about 1460 bp (Fig. 4-A). Meanwhile, double PCR was performed for the amplification of *FliC* and *invA* in the nine isolated strains, and target bands of approximately 588 bp and 175 bp were obtained for both genes (Fig. 4-B), which was consistent with the expected size. Amplification of the *FljB* gene was performed on nine isolates and a target band of approximately 1195 bp was obtained (Fig. 4-C). In our study, the isolated strains were identified through medium screening, bacterial Gram staining identification, and 16 S rRNA, *invA*, *FliC* and *FljB* genome sequence analysis demonstrated that all nine strains isolated from the aborted foal tissues and mare vaginal swabs were *S. abortus equi*. Lactose fermentation capacity is a biochemical characteristic used to distinguish Salmonella longum from other Enterobacteriaceae, however most Salmonella enterica do not contain lactose fermentation genes [18]. colonies of enteropathogenic bacteria on SS agar plates cannot break down lactose and are colorless or yellowish. However, this test showed the colonies of the isolated strains on SS agar plates were transparent colonies, instead of the expected colonies with a black center, due to the presence of unfermented lactose. Therefore, the identification of bacteria cannot rely solely on microbial culture methods and must be complemented by genomic and molecular biology methods for the further accurate identification of microbial species [19]. The final identification of *S. abortus equi* was performed with PCR and positive amplification and nucleotide sequence comparison of specific primers *invA* and *FliC* based on molecular biology.

**Fig. 5:**
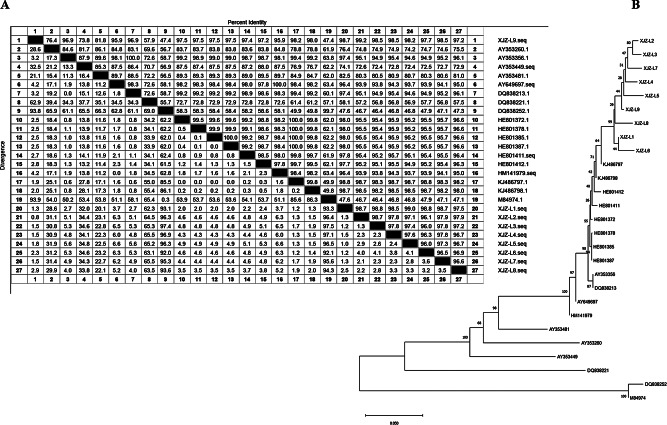
Evolutionary genetic analysis. (**A**) Similarity comparison of *FliC* nucleotide between isolated strains and reference strains; (**B**) Phylogenetic analysis of *FliC* nucleotide isolated strains

The similarity analysis of *FliC* in all nine isolated strains exhibited that the nucleotide sequences of the isolated strains were more than 99% similar to the horse abortus strains in GenBank. The homology analysis of *FliC* in any two isolated *S. abortus equi* with the sequences registered in GenBank was performed with DNASTAR and MEGA7 software. As exhibited in Fig. 5A, the similarity of nucleotide sequences of *FliC* between *S. abortus equi* isolated from Ili, Xinjiang, and other isolated strains of Salmonella ranged from 46.8 to 98.2%. The sequence of the isolated strains in Ili, Xinjiang, had a higher similarity of 98% with that of isolated strains from Ireland, as well as the highest similarity with that of the Chinese isolated strained, which was higher than 98%, consistent with the results of similarity analysis (Fig. 5B). Information on the reference strains is listed in Supplementary Table 8. Li et al. [20] performed the sequence analysis of *FliC* in *S. abortus equi* of horse origin in Xinjiang and observed that the isolated strains had close genetic relationship with *S. abortus equi* (HE801371, HE801374, and HE801378) from Ireland and *salmonella enteritidis* HE801385. Cheng et al., [21] concluded that the *FliC* gene is highly homologous and well conserved within the same species-genus in *salmonella enteritidis*. In our research, the amplification and phylogenetic analysis of *FliC* in *S. abortus equi* revealed some evolutionary differences among the nine isolated strains, among which the isolated strain XJZ-L6 had relatively close genetic relationship with the Chinese isolated strains China-KJ486797 and China-KJ486798 but had distant relationship with XJZ-L2, indicating that the bacteria mutated in the evolutionary process or due to individual differences in the species.

### Results of the bacterial growth curve

**Fig. 6:**
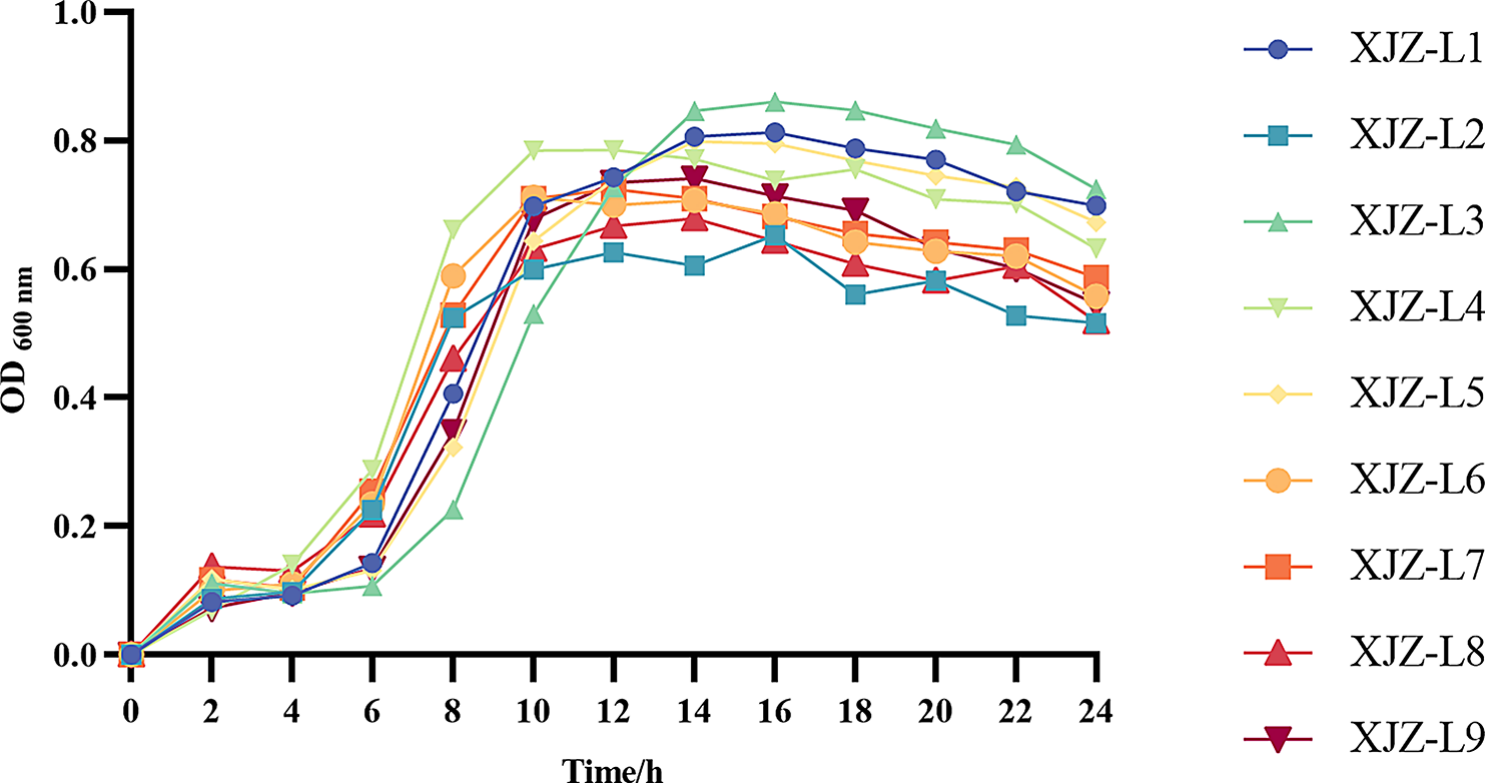
24 h Growth curve of 9 *Salmonella* strains

The OD600 values of the nine isolated strains peaked at 10–16 h, respectively, and all of the nine isolated strains entered the logarithmic growth period after 4 h. Among them, XJZ-L4 had the fastest growth rate, reached the plateau period at 10 h and had a higher number of bacteria, while XJZ-L3 had a slower growth rate, entered the plateau period at 14 h and then the growth rate declined slowly at 18 h. The remaining growth characteristics of the bacteria were similar, but there was also some difference. The growth curves of all nine isolated strains basically conformed to the S-shaped growth curve model (Fig. 6).

### Results of drug sensitivity test

The drug sensitivity test results (Table 3 and Supplementary Fig. 1) revealed that the isolated strains were sensitive to some drugs, including some β-lactams (amoxicillin, penicillin, and ampicillin), cephalosporins (cefuroxime, cefoxitin sodium, methicillin, and ceftriaxone sodium), quinolones (ciprofloxacin, levofloxacin, norfloxacin, and enrofloxacin), aminoglycosides (gentamicin and streptomycin), and some tetracyclines (doxycycline and tetracycline) but insensitive to and develop high resistance to macrolides (clarithromycin and clindamycin), sulfonamides (sulfamethoxazole), and rifampicin.

**Table 3:** Drug sensitivity test results of isolated strains

Serial number	Drugs	Judgment criteria (unit mm)			Antibacterial circle diameter (mm)		XJZ-L2 Sensitivity	XJZ-L6 Sensitivity
		R	I	S	XJZ-L2	XJZ-L6		
1	Amoxicillin	≤ 12	13–14	≥ 15	29.93	29.64	S	S
2	Cefuroxime	≤ 14	15–17	≥ 18	21.02	19.87	S	S
3	Ciprofloxacin	≤ 15	14–20	≥ 21	30.23	27.96	S	S
4	Clarithromycin	≤ 16	17–20	≥ 21	8.01	9.21	R	R
5	Clindamycin	≤ 15	16–18	≥ 19	6.21	6.23	R	R
6	Doxycycline	≤ 14	15–18	≥ 19	17.21	18.23	I	I
7	Levofloxacin	≤ 13	14–16	≥ 17	31.61	31.23	S	S
8	Gentamicin	≤ 12	13–14	≥ 15	19.92	19.67	S	S
9	Cefoxitin	≤ 14	15–17	≥ 18	26.81	28.32	S	S
10	Penicillin	≤ 19	20–25	≥ 26	13.21	12.73	R	R
11	Tetracycline	≤ 18	19–22	≥ 23	25.51	24.67	S	S
12	Sulfamethoxazole	≤ 12	13–14	≥ 15	8.01	7.23	R	R
13	Rifampicin	≤ 12	13–14	≥ 15	8.74	7.59	R	R
14	Norfloxacin	≤ 12	13–16	≥ 17	25.61	25.47	S	S
15	Trimethoprim	≤ 10	11–15	≥ 16	23.91	23.28	S	S
16	Ampicillin	≤ 13	14–16	≥ 17	23.61	21.47	S	S
17	Enrofloxacin	≤ 12	13–16	≥ 17	32.93	32.58	S	S
18	Ceftriaxone Sodium	≤ 13	14–20	≥ 21	27.54	27.67	S	S
19	Streptomycin	≤ 11	12–14	≥ 15	18.19	19.21	S	S

*Note* “S” is Sensitive; “R” is Resistance; “I” is Intermediate

In the study of Pu et al., [22] the resistance phenotypes of two strains of *S. abortus equi* in donkeys from Xinjiang unraveled that the isolated strains were resistant to more than nine antimicrobial drugs and had high resistance to several antimicrobial drugs widely used in clinical practice, such as β-lactams, tetracyclines, and sulfonamides, with multi-drug resistance. Zhu et al. [23] analyzed drug sensitivity of *S. abortus equi* isolated from donkeys of a large-scale donkey farm in Shandong and noted that the isolates were sensitive to 11 commonly used clinical antibiotics, illustrating no antibiotic abuse on this farm. By analyzing sensitivity of nine strains of *S. abortus equi* from donkeys to 11 clinically used antimicrobial drugs, Sun et al. [24] found that the nine strains had a narrow resistance spectrum and were resistant to amoxicillin and streptomycin. In our study, the isolated strains were resistant to 5 antibiotics, sensitive to 13 antibiotics, and intermediary to 1 antibiotic, indicating that drug use rules should be strictly followed and alternative antibiotics should be actively sought to reduce over-reliance on antibiotics in the clinic.

### The tertiary structure prediction of *FliC* protein in nine strains of *S. abortus equi*

**Fig. 7:**
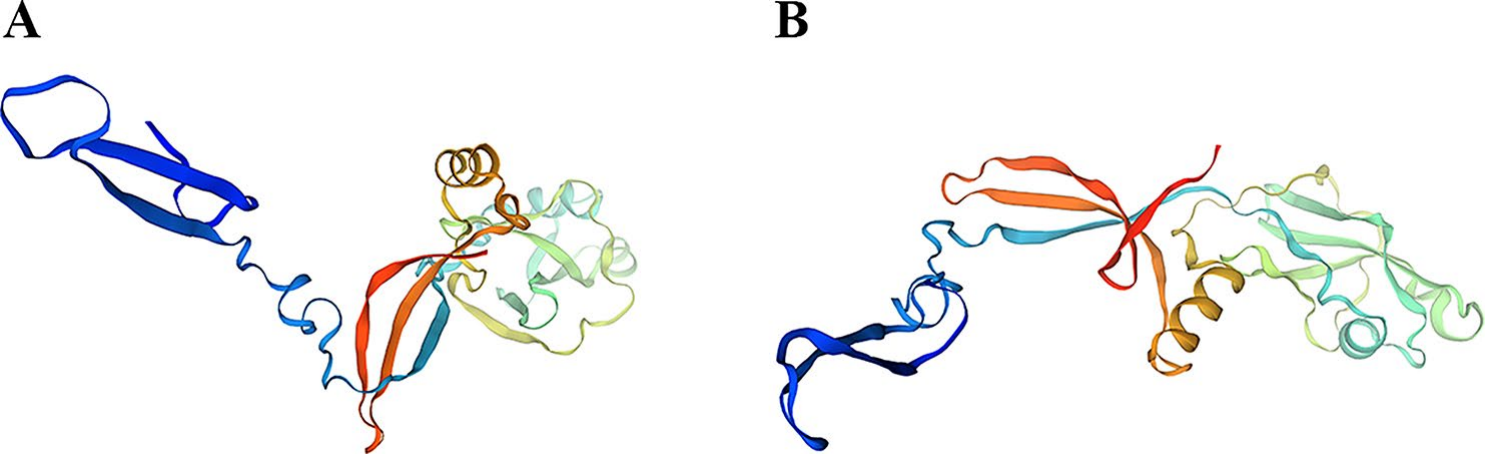
Prediction of the tertiary structure of *Salmonella abortus FliC* protein in horses. (**A**) XJZ-L1-4、XJZ-L6、XJZ-L9; (**B**)XJZ-L5、XJZ-L7-8

The protein tertiary structure of *FliC* protein in the nine isolated *S. abortus equi* strains was predicted, which demonstrated that the structure of *FliC* protein was consistent among six strains, XJZ-L1-4, XJZ-L6, and XJZ-L9, and among three strains, XJZ-L5 and XJZ-L7-8 (Fig. 7).

*Salmonella* can be transmitted through multiple routes, and which is associated with stillbirth, preterm delivery, and chorioamnionitis in addition to intestinal symptoms [25–28]. The known causative factors for equine abortion disease, such as environmental stress, site changes, or changes in forage, may facilitate hormonal imbalances in pregnant horses, which may lead to uterine relaxation and enhance the penetration of pathogenic microorganisms [29]. Some researchers observed that some horses were natural carriers of *S. abortus equi* and that the rise in the number of vaginal infections in mares was fueled by successive abortions and horizontal transmission of the causative agent [30]. Therefore, vaccination against *S. abortus equi* should be performed on a large scale to prevent the transmission of *S. abortus equi*.

## Conclusion

By collecting 971 serum samples from horses in areas of intensive horse breeding in the Ili and Bayingol regions, Xinjiang, we found that *S. abortus equi* infections were prevalent in Xinjiang and that the infection rate of *S. abortus equi* in horses varied significantly (*P* < 0.05) by region, breed, gender, and age. In this study, nine strains were isolated from 65 samples of fetal visceral tissues and vaginal swabs of aborted mares (an isolation rate of 14%) and named XJZ-1-9. All nine isolated strains were identified as *S. abortus equi* with nucleotide sequence comparison, similarity analysis, and phylogenetic tree construction. The isolated strains exhibited high resistance to clarithromycin, clindamycin, penicillin, sulfisoxazole, and rifampicin drugs. Conclusively, our study provides investigation data for the research, prevention, and control of *S. abortus equi* infections in Xinjiang.

### Electronic supplementary material

Below is the link to the electronic supplementary material.

Supplementary Material 1

## Data Availability

The datasets presented in this study can be found in online repositories. The names of the repository/repositories and accession number(s) can be found in the article/Supplementary material.
