# Supplementary material for: Serological investigation and isolation of Salmonella abortus equi in horses in Xinjiang
Source: BMC Vet Res. 2024 Mar 15;20:103. doi: 10.1186/s12917-024-03955-7 (PMC10941388; doi:10.1186/s12917-024-03955-7)
Supplement: Supplementary file 1 — Supplementary Material 1 [file 12917_2024_3955_MOESM1_ESM.docx]

Serological investigation and isolation of *Salmonella abortus equi* in horses in Xinjiang

# Materials and methods

## Reagent

The *Salmonella abortus* ELISA kit was purchased from Harbin Guosheng Biological Co. Ltd. (NEE27100); DNA Marker 2000 (CW0623M), Taq DNA polymerase (CW0690M), bacterial genomic DNA extraction kit (CW0552S), agarose gel DNA recovery kit (CW2302M) were purchased from Kangwei Century Biotechnology Co;

## Instruments

The enzyme marker (Spectra Max Plus384) was purchased from MD, USA; the TPro fessional PCR instrument was purchased from Biometra; the DYY-6C electrophoresis instrument was purchased from Beijing Liuyi Instrument Factory; and the BIO RAD gel imaging system was purchased from MODEL: Universal Hood II.

## Sample collection

42 samples of aborted foal tissues and 23 mare vaginal swabs. Pathogens were isolated and identified after the collection of organ samples (including 7 heart tissues, 6 liver tissues, 5 spleen tissues, 7 lung tissues, 7 kidney tissues, 5 umbilical cord tissues, 5 urinary membrane tissues, and 23 vaginal swabs) through dissection.

# Supplementary Figures and Tables

## Supplementary Figures
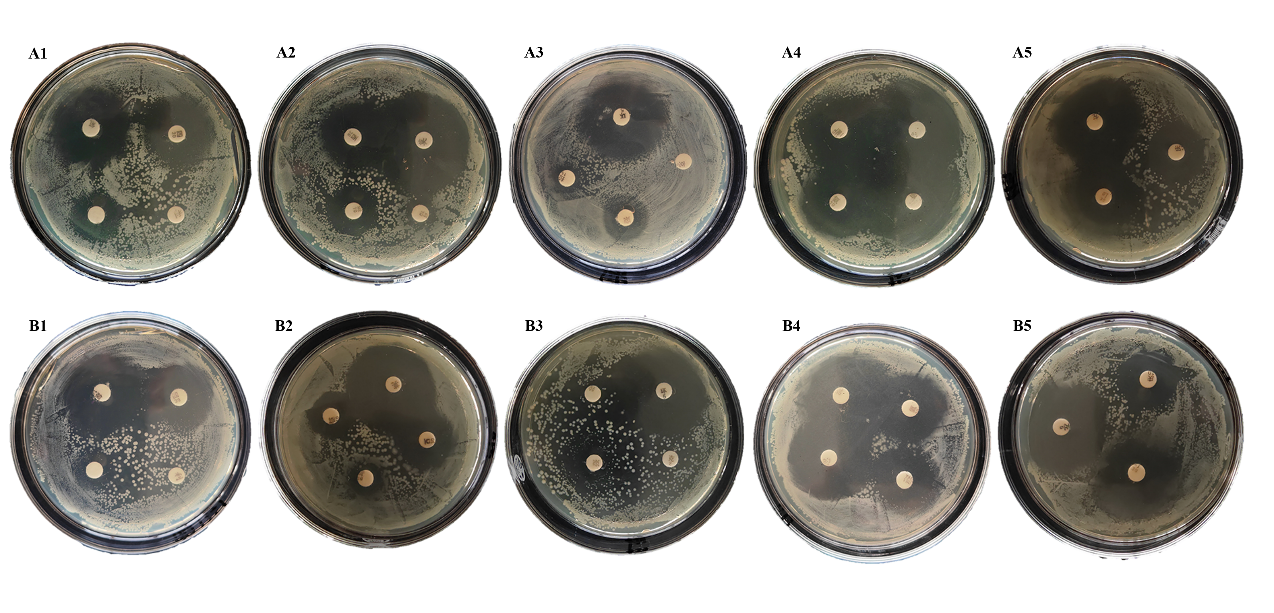


**Supplementary Figure 1.** **Graph of drug sensitivity test results**

(A) A1-5 is the drug sensitivity result of XJZ-L2 strain; (B) B1-5 is the drug sensitivity result of XJZ-L6 strain

## Supplementary Table

**Supplementary Table 1 Results of cardinality test for *S. abotus equi* at different genders**

| **Region** | **Positive** | **Negative** | **Total** | **Positive rate /（%）** | **X^2^** | ***P-value*** |
| --- | --- | --- | --- | --- | --- | --- |
| Yili | 153 | 373 | 526 | 29.09 | 46.458 | 0.001 |
| Korla | 50 | 395 | 445 | 11.24 |  |  |

**Supplementary Table 2 The detection results of** ***S. abotus equi* serum antibodies in different egions**

| Region | County | Sampling Place | | Variety/Breed | Sample No F/M | *S. abotus equi* Positive No F/M |
| --- | --- | --- | --- | --- | --- | --- |
| Ili | Zhaosu | A farms  B farms  C farms | | YL  SBT  HB  YL  SBT  HB  YL  SBT | 27/0  193/15  44/1  136/9  2/0  14/0  56/3  17/9 | 2/0  90/9  15/1  15/0  0/0  1/0  17/0  2/1 |
| Bayingol | Hejing | D farms  E farms  F farms  G farms  H farms | | YQ  YQ  YQ  YQ  YQ | 83/72  62/41  73/18  47/6  39/4 | 16/10  7/0  9/2  3/0  2/1 |
|  |  | |  | Total | 793/178 | 179/24 |

Note: F, female; M, male; YL, Ili; SBT, Self-bred Thoroughbred horse; HB, Half-bred horse; YQ, Yanqi horse

**Supplementary Table 3 Results of two-by-two comparison of the cardinality test for different species of horses *S. abotus equi***

| **Comparison group** | **Positive** | **Negative** | **Total** | **Positive rate /（%）** | **X^2^** | ***P-value*** |
| --- | --- | --- | --- | --- | --- | --- |
| SBT | 102 | 134 | 236 | 43.22 | 4.071 | 0.04 |
| HB | 17 | 42 | 59 | 28.81 |  |  |
| YL | 34 | 197 | 231 | 14.72 | 1.695 | 0.193 |
| YQ | 50 | 395 | 445 | 11.24 |  |  |
| SBT | 102 | 134 | 236 |  | 45.943 | 0.001 |
| YL | 34 | 197 | 231 |  |  |  |
| SBT | 102 | 134 | 236 |  | 90.990 | 0.001 |
| YQ | 50 | 395 | 445 |  |  |  |
| HB | 17 | 42 | 59 |  | 6.442 | 0.01 |
| YL | 34 | 197 | 231 |  |  |  |
| HB | 17 | 42 | 59 |  | 13.964 | 0.001 |
| YQ | 50 | 395 | 445 |  |  |  |

**Supplementary Table 4 The detection results of *S. abotus equi* serum antibodies in different age**

| Age | Sample No F/M | *S. abortus* Positive No F/M | *S. abortus* Positive No F/M (%) |
| --- | --- | --- | --- |
| 0＜Age≤1 | 17/13 | 12/9 | 40.00/30.00 |
| 1＜Age≤3 | 110/45 | 15/2 | 9.68/1.29 |
| 3＜Age≤15 | 624/119 | 134/13 | 18.04/1.75 |
| Age＞15 | 42/1 | 18/0 | 41.86/0 |
| Total | 793/178 | 179/24 | 18.44/2.47 |

**Supplementary Table 5 Results of cardinality test for *S. abotus equi* at different ages**

| **Age group** | **Comparison group** | **Positive** | **Negative** | **Total** | **Positive rate /（%）** | **X^2^** | ***P-value*** |
| --- | --- | --- | --- | --- | --- | --- | --- |
| Juvenile | 0＜Age≤1 | 21 | 9 | 30 | 70.00 | 53.666 | 0.001 |
| Youth | 1＜Age≤3 | 17 | 138 | 155 | 10.97 |  |  |
| Adult | 3＜Age≤15 | 147 | 587 | 743 | 19.25 | 11.577 | 0.001 |
| Old | Age＞15 | 18 | 25 | 43 | 41.86 |  |  |
| Juvenile | 0＜Age≤1 | 21 | 9 | 30 |  | 41.959 | 0.001 |
| Adult | 3＜Age≤15 | 147 | 587 | 743 |  |  |  |
| Juvenile | 0＜Age≤1 | 21 | 9 | 30 |  | 5.623 | 0.018 |
| Old | Age＞15 | 18 | 25 | 43 |  |  |  |
| Youth | 1＜Age≤3 | 17 | 138 | 155 |  | 6.982 | 0.008 |
| Adult | 3＜Age≤15 | 147 | 587 | 743 |  |  |  |
| Youth | 1＜Age≤3 | 17 | 138 | 155 |  | 5.623 | 0.018 |
| Old | Age＞16 | 18 | 25 | 43 |  |  |  |

**Supplementary Table 6 Results of cardinality test for *S. abotus equi* at different genders**

| **Genders** | **Positive** | **Negative** | **Total** | **Positive rate /（%）** | **X^2^** | ***P-value*** |
| --- | --- | --- | --- | --- | --- | --- |
| Female | 179 | 614 | 793 | 22.57 | 7.263 | 0.007 |
| Male | 24 | 154 | 178 | 13.48 |  |  |

**Supplementary Table 7 Results of cardinality test for *S. abotus equi* at different genders**

| **Feeding models** | **Positive** | **Negative** | **Total** | **Positive rate /（%）** | **X^2^** | ***P-value*** |
| --- | --- | --- | --- | --- | --- | --- |
| Scale farms | 150 | 388 | 538 | 27.88 | 35.494 | 0.001 |
| Free-range farms | 53 | 380 | 433 | 12.24 |  |  |

**Supplementary Table 8 Reference strain information**

| **Name** | **Genbank Login Number** | **Source** |
| --- | --- | --- |
| *Salmonella bispebjerg (S. bispebjerg)* | HE801387 | Ireland |
| *Salmonella enterica (S. enterica)* | HE801385 | Ireland |
| *Salmonella arechavaleta (S. arechavaleta)* | HE801411 | Ireland |
| *Salmonella abortion equine (S. abortus equi)* | HE801378 | Ireland |
| *Salmonella paratyphi (S. paratyphi)* | DQ838213 | Australia |
| *Salmonella enterica (S. enterica)* | AY353356 | USA |
| *Salmonella enterica (S. enterica)* | HM141979 | USA |
| *Salmonella fulica (S. fulica)* | HE801372 | Ireland |
| *Salmonella bongori (S. bongori)* | AY353260 | USA |
| *Salmonella typhi (S. typhi）* | DQ838221 | Australia |
| *Salmonella enteritidis (S. enteritidis)* | M84974 | USA |
| *Salmonella pullorum (S. pullorum)* | DQ838252 | Australia |
| *Salmonella enterica (S. enterica)* | AY353449 | USA |
| *Salmonella enterica (S. enterica)* | AY353481 | USA |
| *Salmonella enterica (S. enterica)* | AY649697 | USA |
| *Salmonella enterica (S. enterica)* | HE801412 | Ireland |
| *Salmonella enterica (S. enterica)* | KJ486797 | China |
| *Salmonella enterica (S. enterica)* | KJ486798 | China |
| XJZ-L1 | OR565965 | China |
| XJZ-L2 | OR565966 | China |
| XJZ-L3 | OR565967 | China |
| XJZ-L4 | OR565968 | China |
| XJZ-L5 | OR565969 | China |
| XJZ-L6 | OR565970 | China |
| XJZ-L7 | OR565971 | China |
| XJZ-L8 | OR565972 | China |
| XJZ-L9 | OR565973 | China |

# Footnotes

1. http://www.ncbi.nlm.nih.gov/BLAST

2. <https://swissmodel.expasy.org/>

3. https://www.jianguoyun.com/p/DWaB0w8Q3sfjCxi4to8FIAA
